# Huangqin Tang Interference With Colitis Associated Colorectal Cancer Through Regulation of Epithelial Mesenchymal Transition and Cell Cycle

**DOI:** 10.3389/fphar.2022.837217

**Published:** 2022-04-06

**Authors:** Xuran Ma, Dunfang Wang, Xue Feng, Yaqing Liu, Jia Li, Weipeng Yang

**Affiliations:** ^1^ Institute of Chinese Materia Medica, China Academy of Chinese Medical Sciences, Beijing, China; ^2^ School hospital, Tsinghua University, Beijing, China

**Keywords:** colitis-associated colorectal cancer, azoxymethane/dextran sodium sulfate model, proteomic, G1/S, epithelial mesenchymal transition

## Abstract

**Background:** Although the exact molecular mechanisms of colitis-associated colorectal cancer are not fully understood, the chronic inflammation was positively correlated with tumorigenesis. The traditional Chinese medicine botanical formulation Huangqin Tang has significant anti-inflammatory effects. We investigated whether HQT can ameliorate the progression of inflammation to cancer through its anti-inflammatory effects by using relevant predictions and experiments.

**Methods:** We used the azoxymethane/dextran sodium sulfate method to induce the mice colitis-associated colorectal cancer model. After preventive administration of Huangqin Tang to the mice model, colonic tissues were taken for quantitative proteomic analysis of tandem mass tags, and the proteomic results were then experimentally validated using the molecular biology approach.

**Results:** Proteomic screening revealed that the effect of the mechanism of Huangqin-Tang on the colitis-associated colorectal cancer mice model may be related to infinite replication which demonstrated abnormal G1/S checkpoint and epithelial mesenchymal transition acceleration. The levels of inflammatory factors such as interleukin-1*α*, interleukin-1*β*, interleukin-6, and tumor necrosis factor-*α* were significantly reduced in colitis-associated colorectal cancer mice treated with Huangqin Tang; the aberrant expression of G1/S checkpoint-associated sites of cell cycle protein-dependent kinase 4, D1-type cyclins, and dysregulation of related sites of the WNT pathway which are most related to the acceleration of the epithelial mesenchymal transition process including WNT3A, *β*-catenin, E-cadherin, and glycogen synthase kinase 3*β* has been improved.

**Conclusion:** Reducing inflammation and thus inhibiting the progression of colitis-associated colorectal cancer by using Huangqin-Tang is effective, and the mechanism of action may be related to the inhibition of uncontrolled proliferation during tumorigenesis. In the follow-up, we will conduct a more in-depth study on the relevant mechanism of action.

## Introduction

Colitis-associated colorectal cancer (CAC) is prevalent worldwide and has become a major health problem for public; chronic inflammations are highly involved in the initiation and progression of CAC. Chronic inflammation is a physiological response that is caused by sustained tissue trauma, and this feature causes cellular changes and immune responses that result in repair of the injured tissue and cellular proliferation at the site of the damaged tissue (Grivennikov et al., 2010). The proliferation, differentiation, and apoptosis of intestinal mucosal epithelial cells are maintained in a dynamic balance, and the loss of epithelial cells has to be compensated by increased epithelial proliferation as a repair mechanism which may finally be uncontrolled leading to CAC. A meta-analysis has recently shown that the cumulative incidence of CAC is 0.1, 2.9, and 6.7% after 10, 20, and 30 years (Choi et al., 2015), respectively. Therefore, treating the inflammatory causes is always important.

The proliferation of eukaryotic cells is typically divided into four sequential phases of cell cycle: G1 (pre-DNA synthesis), S (DNA synthesis), G2 (pre-division), and M (cell division) (Sherr, 1996). During the interphase, the G1/S transition is a severe restriction point, resulting in one of the three destinies for the cell: continue cycling, exit active proliferation, or enter a quiescent (G0) state (Chen and Pan, 2017). Cyclin-dependent kinases (Cdks) have pivotal roles in regulating the cell cycle mechanism and to a large degree govern cellular transitions from growth phases (G1 and G2) into phases associated with DNA replication (S) and mitosis (M) (Hamilton and Infante, 2016). The abrogation of the G1/S checkpoint or upregulation of the D1-type cyclins (Cyclin D1, Ccnd1)/cyclin-dependent kinases (Cdk4) pathway provides an obvious advantage to cancer cells in terms of proliferation and survival.

The epithelial mesenchymal transition (EMT) is a highly dynamic process of interconversion of epithelial cells into quasi-mesenchymal cells, which is known to be essential for embryogenesis, wound healing, and malignant progression (Nieto et al., 2016). In the context of neoplasias, EMT confers increased tumor initiation and metastatic potential to cancer cells (Dongre and Weinberg, 2019). Normally, the cells that form the epithelial layer in various tissues of the body display apical–basal polarity and are held together laterally by tight junctions and adherens junctions, the latter formed by the cell surface epithelial cadherin (E-cadherin, Cdh1) molecules. This organization is essential for the structural integrity of epithelial cells. The canonical WNT pathway has long been considered a key activator of EMT (Savagner, 2001). Cdh1 is a negative regulator of the canonical WNT pathway and in the context of wound healing, *β*-catenin (Ctnnb1) is recruited into a destruction complex that contains adenomatous polyposis coli (APC) protein and framework protein axin (Axin), which facilitates the phosphorylation of Ctnnb1 by casein kinase 1(CK1) and then glycogen synthase kinase 3 beta (Gsk3*β*) (Arwert et al., 2012). This leads to the accumulation of Ctnnb1 in the cytoplasm after ubiquitination and proteasomal degradation and then translocation into the nucleus where they bind to WNT ligands and response to the activation of the WNT pathway (Gonzalez and Medici, 2014). After that, Cdh1 expression is inhibited which promotes EMT and enhances the remodeling of the extracellular matrix (ECM) into a matrix with different composition and properties.

Recently, natural products have been widely used in preventing and treating gastrointestinal disorders. Traditional Chinese medicines (TCMs) and extracts have shown various beneficial treatment effects including bacteriostasis and anti-inflammation (Chen et al., 2015). Among them, Huangqin-Tang (HQT) is a well-known classic botanical formulation which is derived from the Chinese medicine book “Shang-Han-Lun.” Pharmacokinetic studies on multiconstituents in HQT by the validated HPLC method showed that the major effective components of the four botanical drugs are flavonoids (e.g., wogonin, baicalein, and oroxylin-A) (Li et al., 2016). These constituents have a wide range of pharmacological characteristics, such as anti-inflammation, analgesic, and immunomodulatory (Wang et al., 2020).

The goal of this study was to explore whether HQT can suppress the tumorigenesis and malignancy development of CAC *via* its anti-inflammatory effects. Azoxymethane (AOM)/dextran sodium sulfate (DSS)-induced CAC can be utilized in combination with HQT in order to determine and validate novel therapeutic targets. We used proteomic techniques to select prime targets followed by molecular biology techniques to examine their protein and gene expression. Thus, this study provides new insights into discovery of the critical therapeutic regulators of CAC, making HQT that emerged as a “low toxic-high pharmacological effective” agent for prevention or treatment of CAC.

## Materials and Methods

### Chemicals

Azoxymethane (Sigma, United States), dextran sulfate sodium (36–50 kDa, MP Biomedicals, United States), phosphate-buffered saline (Hyclone, United States), PageRuler Plus Prestained Protein Ladder (ThermoFisher, United States), TMT^®^ Mass Tagging Kits and Reagents (ThermoFisher, United States), dithiothreitol (Sigma, United States), iodoacetamide (Sigma, United States), sodium dodecyl sulfate (Sigma, United States), urea (Sigma, United States), pancreatin (Promega, China), ammonium bicarbonate (Sigma, United States), UltraPure water (ThermoFisher, United States), triethylammonium bicarbonate buffer (Sigma, United States), acetonitrile (ThermoFisher, United States), formic acid (ThermoFisher, United States), acetone (ThermoFisher, United States), ProteoMiner Low-Abundance Protein Enrichment Kit (Bio-Rad, United States), and trifluoroacetate (Sigma, United States) were used in this study.

### Animals

Balb/c male mice (18–20 g) were obtained from the Laboratory Animal Center of the National Institutes for Food and Drug Control (Production license no. SCXK 2017-0005). All mice were housed at 23 ± 1.5°C. The animal experiment process was conducted in accordance with the ethical guidelines for local animal care and usage.

### Preparations of Huangqin-Tang

*Sculellaria baicalensis* Georgi, *Paeonia lactiflora* Pall, *Glycyrrhiza uralensis* Fisch, and *Ziziphus jujuba* Mill (weight ratio 3:2:2:3) were weighed and mixed. The decoction was boiled twice with ddH_2_O at 100°C in the ratio of (1:10, w/v) (1:8, w/v) for 1 h each time. The filtrate of both batches was collected and combined. Then, the filtrate was dried completely under a reduced pressure environment to obtain HQT extract. The final concentration of the extract was 1 g ml^−1^. The extraction yield was 39.69%.

### Azoxymethane/Dextran Sodium Sulfate Model Construction

**FIGURE 1:**
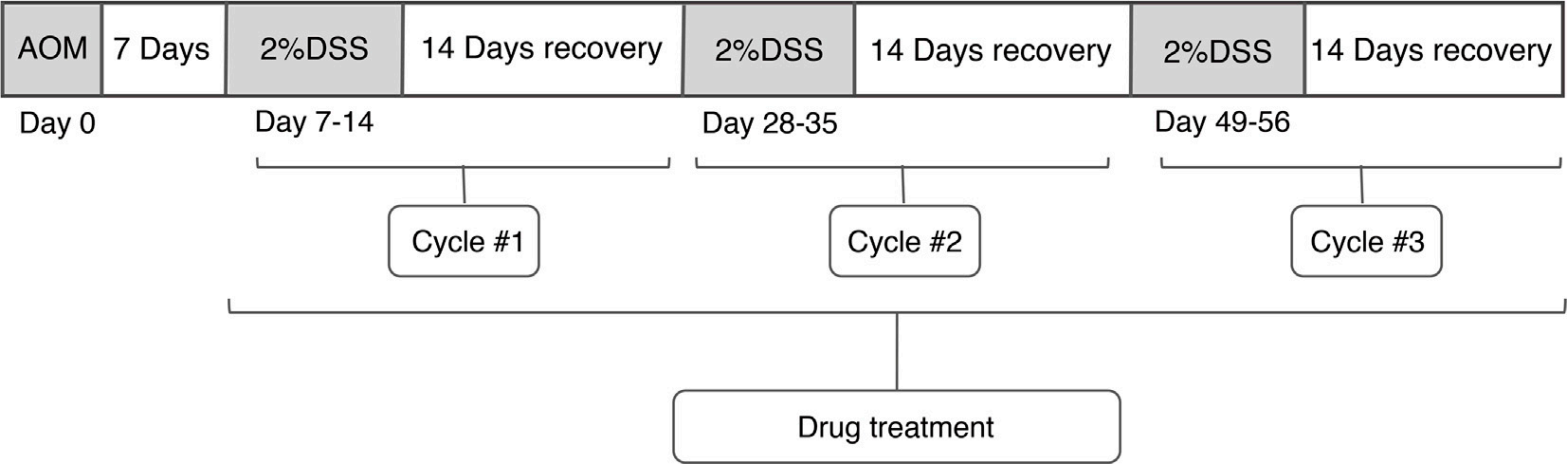
AOM/DSS model construction cycle.

Injected azoxymethane (AOM): The mice were weighed, and accurate weights were required in order to ensure uniform dosing of AOM. The volume of AOM was calculated to inject to achieve a dose of 10 g ml^−1^. For example, a 20-g mouse would receive a 200 μl injection of 1 mg ml^−1^ AOM solution. HQT was weighed, and mice were intragastric administrated per day. Dextran sodium sulfate (DSS) -dH_2_O cycle was performed 7 days after AOM injection and prepared a total of 300–400 ml of 2% DSS to be used per mouse cage. The mice were left with 2% DSS in drinking water for 7 days, followed by 14 days of recovery by replaced 2% DSS with dH_2_O. This “7 days 2% DSS–14 days dH_2_O” cycle was looped three times (Figure 1). The mice were weighed daily, and the treatment groups were gavaged with HQT (20 g kg^−1^) until the end of the experiment. The specimen was collected by euthanizing the mice at the end of the last recovery. The abdomen was pinched and pulled up at the midline using dissection forceps, and an incision was cut in the skin of the abdominal side, and then the incision was extended to the xyphoid process at the midline and to the costal margins bilaterally. The abdominal muscle tissue was gently pulled away, and then an incision was made in the peritoneal tissue carefully and continued to cut away to expose the intestines. The entire length of the colon from any mesenteric connective and fat tissue was unraveled. Once the cecum was identified, it was cut through the distal colon including the anus. The colon was flushed with PBS using a 10 -MmL syringe, and then the colon was cut longitudinally.

### Serum Cytokine Detection

After the end of the last recovery, the blood samples were collected by the eyelid method and then centrifuged (3,000 rpm, 15 min) to get the serum. The levels of IL-1*ɑ*, IL-1*β*, TNF-*ɑ*, and IL-6 were detected by using Luminex kits in accordance with the manufacturer’s specifications.

### Histological Examination

The colonic segments which were collected from all groups were embedded and sliced. Then, 4-μm-thick tissue sections were prepared and stained with hematoxylin and eosin (H&E) for histological studies. The degree of colon lesions was compared.

### Tandem Mass Tags Quantitative Proteome

All labeling samples were mixed with equal volume, desalted and lyophilized. Mobile phase A (2%acetonitrile, adjusted pH=10.0 using ammonium hydroxide) and B(98%acetonitrile) were used to develop a gradient elution. The sample was fractionated using a C18 column (Waters BEH C18, 4.6×250mm, 5μm) on a Rigol L3000 HPLC system, the column oven was set as 45°C. The eluates were monitored at UV 214nm, collected for a tube per minute and combined into 10fractions finally. All fractions were dried under vacuum, and then, reconstituted in 0.1%(v/v) formic acid (FA) in water. For transition library construction, shotgun proteomics analyses were performed using an EASY-nLCTM 1200 UHPLC system (ThermoFisher, USA) coupled with a Q ExactiveTM HF-X mass spectrometer (ThermoFisher, USA) operating in the data-dependent acquisition (DDA) mode. 1μg sample was injected into a home-made C18 Nano-Trap column (4.5cm×75μm, 3μm). Peptides were separated in a home-made analytical column (15cm×150μm, 1.9μm), using a linear gradient elution (0‐10 min, 3‐5% B; 10‐30 min, 5‐20% B; 30‐48 min, 20‐40% B; 48‐50 min, 40‐50% B; 50‐53 min, 50‐70% B; 16.5‐19 min, 48‐52% B; 19‐23 min, 52‐95% B; 53‐54 min, 70‐100% B. The total run time was 54 min.) The separated peptides were analyzed by Q ExactiveTM HF-X mass spectrometer (ThermoFisher, USA), with ion source of Nanospray FlexTM (ESI), spray voltage of 2.3kV and ion transport capillary temperature of 320°C. Full scan range from m/z 350 to 1500 with resolution of 60000(at m/z200), an automatic gain control (AGC) target value was 3×106 and a maximum ion injection time was 20ms. The top 40 precursors of the highest abundant in the full scan were selected and fragmented by higher energy collisional dissociation(HCD) and analyzed in MS/MS, where resolution was 45000(at m/z200) for 10plex, the automatic gain control(AGC) target value was 5×104 the maximum ion injection time was 86ms, a normalized collision energy was set as 32%, an intensity threshold was 1.2×105, and the dynamic exclusion parameter was 20s. Analyzed the identification, functional and quantitation of protein and DEP finally.

### Western Blotting Analysis

The protein expression levels were analyzed by Western blotting. The total proteins from mice colonic segments were lysed using RIPA lysis buffer containing 1% 100 mM phenylmethanesulfonyl fluoride and were then quantified using a BCA Protein Assay Kit (Solarbio, China), and thirty micrograms of protein per lane was separated on a 12% SDS-PAGE gel, and the separated proteins were electroblotted onto nitrocellulose membranes. The membranes were blocked for 1 h in 5% nonfat dry milk dissolved in TBST (20 mM Tris pH = 7.4, 137 mM NaCl, 0.5% Tween-20). After blocking, the blots were incubated overnight at 4°C with the specific antibodies for *β*-actin (SantaCruz, United States, Cat#SC-8432), Ccnd1 (CST, United States, Cat# 2978), Cdk4(CST, United States, Cat# 12790), Cdh1 (CST, United States, Cat# 3195), Gsk3*β* (CST, United States, Cat# 12456), WNT3A (CST, United States, Cat# 2721), and Ctnnb1 (CST, United States, Cat# 8480). The secondary anti-rabbit (ZSGB, China, Cat# ZB-5301) and anti-mouse antibodies (ZSGB, China, Cat# ZB-5305) were incubated with the blots at room temperature on the next day. The Immobilon Western Chemilum HRP Substrate (Millipore, United States) was applied to develop the blots and then assayed the intensity of protein bands by Quantity One 1-D analysis software.

### Quantitative Real-Time PCR Analysis

The total RNA from mice colonic segments was extracted using RNA Easy Fast Tissue/Cell Kit (TIANGEN, China) and the total RNA concentration and purity was assessed by using a NanoDrop One Spectrophotometer (ThermoFisher, SA). The single-stranded complementary DNA was synthesized with a reverse transcription reaction using a PrimeScript™ RT reagent Kit Perfect Real Time (TAKARA, Japan). All sequences of primers used for quantitative RT-PCR are shown in (Table 1). The mRNAs were then amplified *via* a LightCycler 96 system (Roche Diagnostics, Swiss) using TB Green^®^Premix Ex Taq™ Ⅱ Tli RNaseH Plus (Takara, Japan) according to the manufacturer’s protocols. The fold changes were analyzed using the *ΔΔ*Ct method.

**TABLE 1:** Primers used for the quantitative PCR.

Gene	Primer sequence 5′→3′
*β-actin*	Forward: 5′-CTA​CCT​CAT​GAA​GAT​CCT​GAC​C-3′
	Reverse: 5′-CAC​AGC​TTC​TCT​TTG​ATG​TCA​C-3′
*Ccnd1*	Forward: 5′-GAG​GCG​GAT​GAG​AAC​AAG​CAG​AC-3′
	Reverse: 5′-GGA​GGG​TGG​GTT​GGA​AAT​GAA​CTT​C-3′
*Cdk4*	Forward: 5′-ATG​CTA​CCT​CTC​GAA​TGA​GCC​A-3′
	Reverse: 5′-ATG​CTA​CCT​CTC​GAA​TGA​GCC​A-3′
*Cdh1*	Forward′-CTCAGAAGACAGAAACGAGACT-3′
	Reverse: 5′-AAC​CAG​GTT​CTT​TGG​AAA​TTC​G-3′
*Ctnnb1*	Forward: 5′-CTG​CTG​TCC​TAT​TCC​GAA​TGT​CTG​AG-3′
	Reverse: 5′-GGC​ACC​AAT​GTC​CAG​TCC​AAG​ATC-3′
*Gsk3β*	Forward: 5′-TGG​TAG​CAT​GAA​AGT​TAG​CAG​A-3′
	Reverse: 5′-CTC​TCG​GTT​CTT​AAA​TCG​CTT​G-3′
*WNT3A*	Forward: 5′-AAG​TAT​GAT​AGC​GCG​GCG​GC-3′
	Reverse: 5′-TTG​CGC​ACA​CAG​TAG​TCC​GG-3′

### Statistical Analysis

All statistical analyses were conducted using SPSS version 18.0 (SPSS Inc, Chicago, IL, United States) and GraphPad Prism software (GraphPad, La Jolla, CA, United States). Our data were expressed as mean ± standard deviation (SD), and Student’s t-test or one-way ANOVA was used for comparison between groups.

### Proteomic Analysis

The resulting spectra from each run were searched separately against the UniProt database by the search engines Proteome Discoverer 2.4 (PD2.4, Thermo). The searched parameters are set as follows: the mass tolerance for precursor ion was 10 ppm, and mass tolerance for product ion was 0.02 Da. Carbamidomethyl was specified as fixed modifications, and oxidation of methionine (M) and TMT plex was specified as dynamic modification. Acetylation, TMT plex, and Met-loss and Met-loss + Acetyl were specified as N-terminal modification in PD2.4. A maximum of two missed cleavage sites were allowed.

In order to improve the quality of analysis results, the software PD2.4 was further filtered for the retrieval results: peptide–spectrum matches (PSMs) with a credibility of more than 99% were considered identified PSMs. The identified protein contains at least one unique peptide. The identified PSMs and protein were retained and performed with FDR no more than 1.0%. The protein quantitation results were statistically analyzed by the t-test. The proteins, whose quantitation was significantly different between experimental and control groups {*p* < 0.05 and |log2FC|>^*^[FC>^*^or FC<^*^(fold change, FC)]}s were defined as differentially expressed proteins (DEPs).

Gene Ontology (GO) and InterPro (IPR) functional analysis were conducted using the InterProScan program against the nonredundant protein database (including Pfam, PRINTS, ProDom, SMART, PROSITE, and PANTHER), and the databases of COG (Clusters of Orthologous Groups) and KEGG (Kyoto Encyclopedia of Genes and Genomes) were used to analyze the protein family and pathway. DEPs were used for volcanic map analysis, cluster heat map analysis, and enrichment analysis of GO, IPR, and KEGG. The probable protein–protein interactions were predicted using the STRING-db server.

## Results

### Huangqin-Tang Ameliorated the Symptoms of Colitis-Associated Colorectal Cancer Mice

**FIGURE 2:**
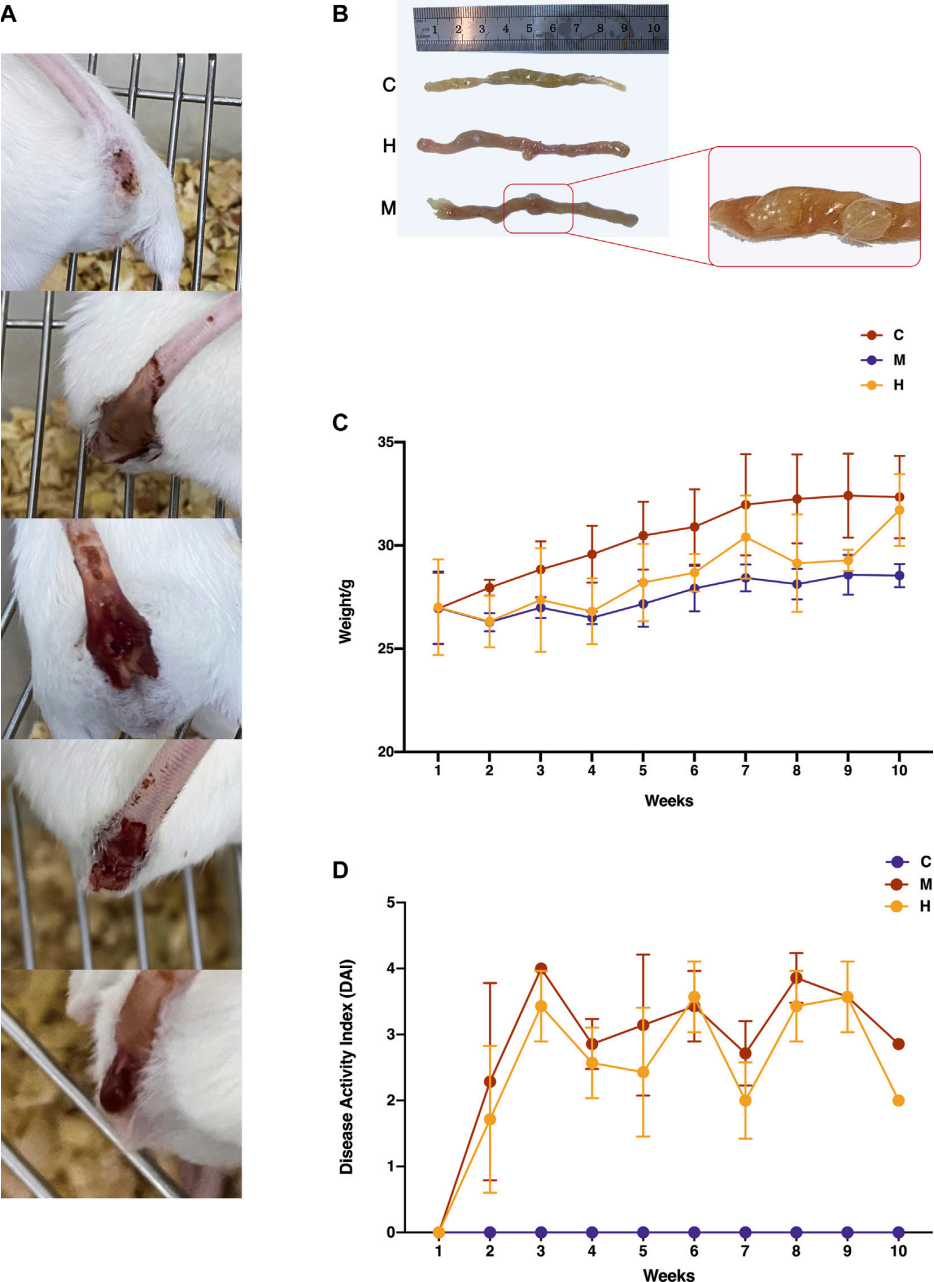
Effect of HQT on AOM/DSS-induced CAC mice. **(A)** Changes in fecal traits of model mice during the induction of AOM/DSS. As the modeling progressed (shown in the figure from top to bottom), mice gradually developed dilute stools, mucus stools, pus, and blood stools and finally detached and developed tumors. **(B)** Changes in colonic tissues of mice during AOM/DSS induction. C: Normal control (daily gavage of equal volume of saline); M: model control (combined AOM/DSS induction, daily gavage of equal volume of saline); and H: HQT control (combined AOM/DSS induction, daily gavage of 20 g kg^−1^ HQT). The number and size of tumors in the HQT group were smaller than those in the model group. **(C)** Body weight changes in mice during AOM/DSS induction. The weight of mice dropped sharply after the first administration of DSS and regained weight after the administration of HQT. **(D)** DAI scores of mice during AOM/DSS induction. The results showed that HQT groups had lower scores than the model group.

The CAC mice induced by AOM/DSS showed severe colitis at the initial stage, with a series of symptoms shown in Figure 2(A), such as diarrhea, apathetic, drowsiness, poor appetite, and weight loss. Then, purulent and blood stools gradually appeared which indicated the development of multiple colon tumors in Figure 2(B). As shown in Figure 2(C)(D), after prolonged HQT exposure, mice’s responses to aforementioned symptoms were all improved significantly, especially their weight gradually increased during the recovery period. The model group mice had no obvious improvement in symptoms.

### Histological Study

**FIGURE 3:**
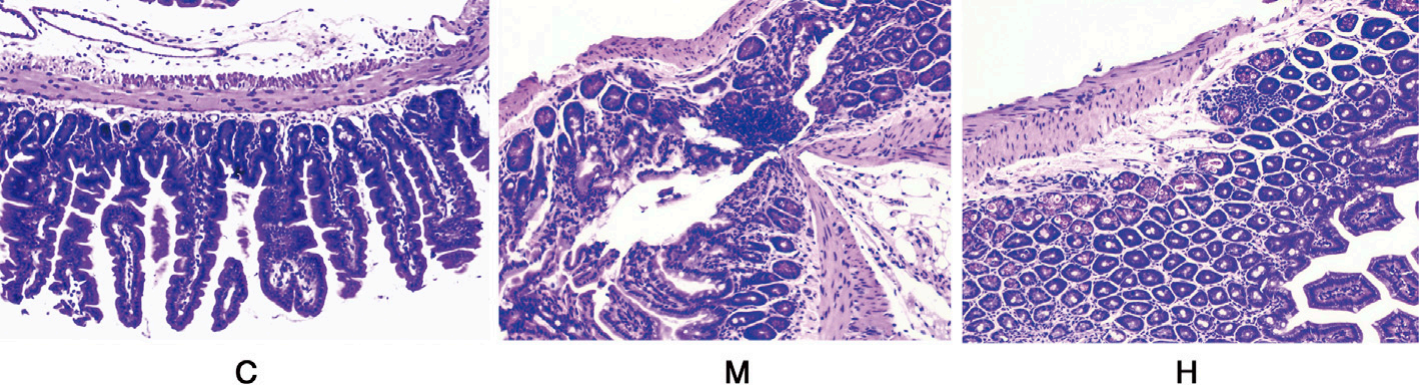
Histological observations of the colon tissues in different groups (HE staining) (10 × 40). C: normal control; M: model control; H: HQT control.

As shown in (Figure 3), the normal mice colon tissues consist of mucosal layer, submucosal layer, lamina propria layer, and plasma layer. The intestinal glands were long and tubular, with a large number of vacuolated cup-shaped cells. Some of the glands in the colon of mice induced with AOM/DSS had lost their circular tubular arrangement and were irregularly shaped. The glandular arrangement was disorganized with heterogenous cells and deep nuclear staining. Also, the luminal surface of the tumor showed high-grade intraepithelial neoplastic changes. After continuous administration of HQT, the structural disruption and disturbance of the colonic crypt and colonic villi in mice were improved, and the degree of inflammatory infiltration was reduced compared with the model group.

### Huangqin-Tang Decreases the Serum Level of Inflammatory Cytokines

**FIGURE 4:**
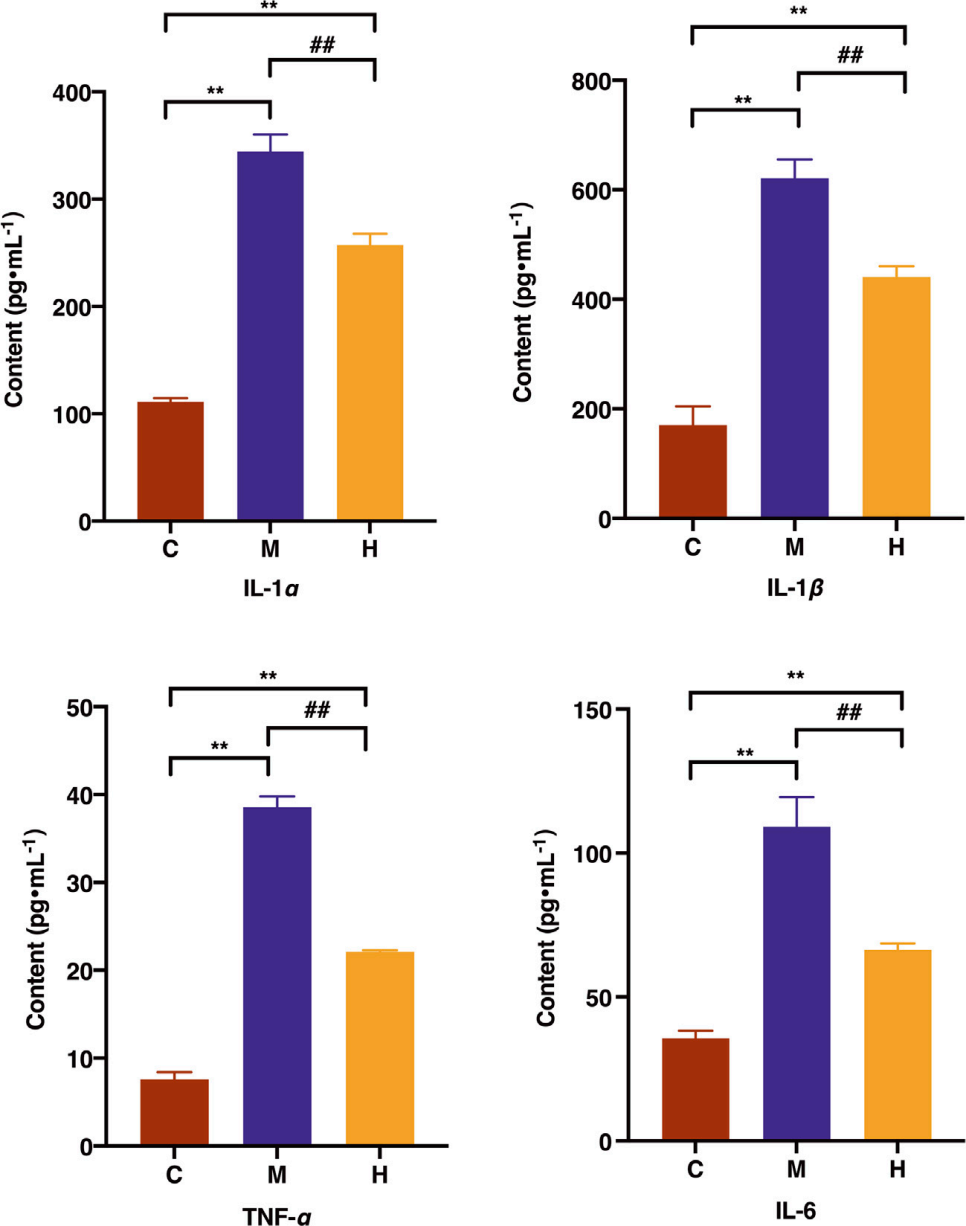
Effects of HQT on the levels of IL-1*ɑ*, IL-1*β*, IL-6, and TNF-*ɑ* after 63 days of oral administration on CAC mice. C: normal control; M: model control; H: HQT control. Data are represented as the mean ± SD (*n* = 10, ¯*x* ± *s*). ^*^ *p* < 0.05, ^**^ *p* < 0.01 vs normal control; ^#^ *p* < 0.05, ^##^ *p* < 0.01 vs. model control.

The levels of pro-inflammatory factors IL-1*ɑ*, IL-1*β*, IL-6, and TNF-*ɑ* in the model group were increased remarkably. In addition, the levels of IL-1*ɑ*, IL-1*β*, IL-6, and TNF-*ɑ* in the HQT group were significantly lower than those in the model group (*p* < 0.01) (Figure 4).

### Diversified Analysis of Proteomics

**FIGURE 5:**
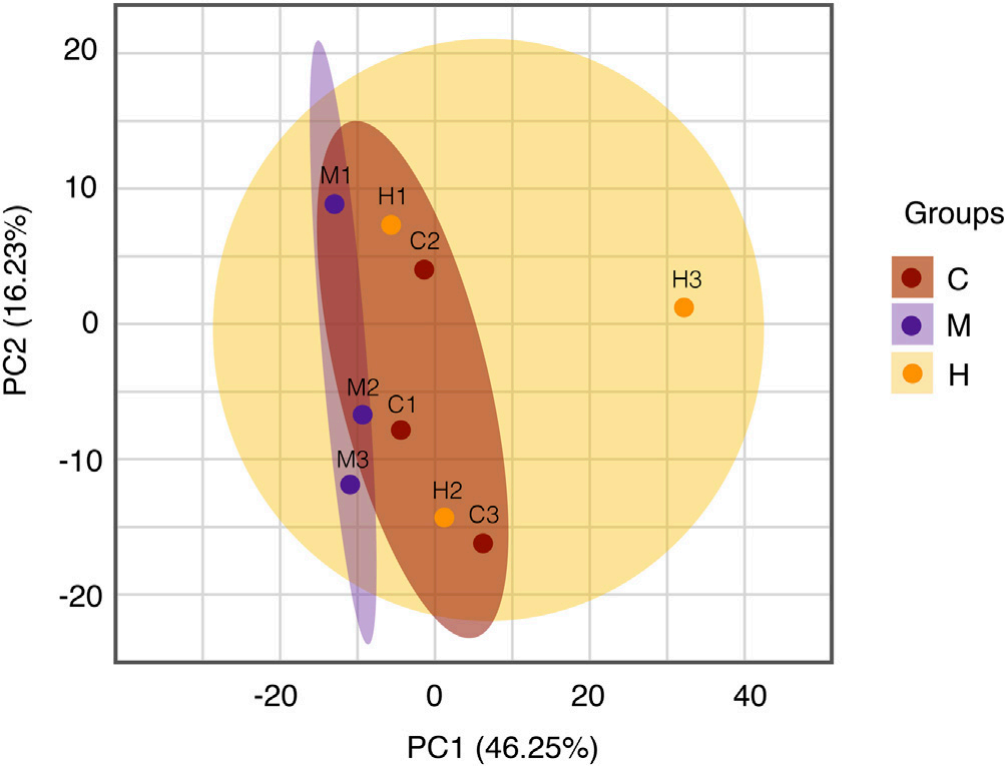
PCA analysis. The central and vertical coordinates PC1 and PC2 indicate the scores of the first and second principal components, respectively, and the color of the scattered dots indicates the experimental grouping of samples. C: normal control; M: model control; H: HQT control, *n* = 3.

Protein quantitative analysis: Colonic tissues data from the normal group, model group, and HQT group were analyzed using unsupervised principal component analysis (PCA). As can be seen from the scores (Figure 5) in which there is some tendency for each group of samples to cluster; the model group(M) is clearly separated from the other groups, and the normal protein expression of mice body was disturbed, and the CAC model can be considered to be successful. Compared with the model group, the HQT group(H) showed a much pronounced trend of clustering to the normal group(C), which indicated that protein expression in mice changed after HQT administration, which suggested that HQT has some regulatory effect on protein expression in CAC mice.

**FIGURE 6:**
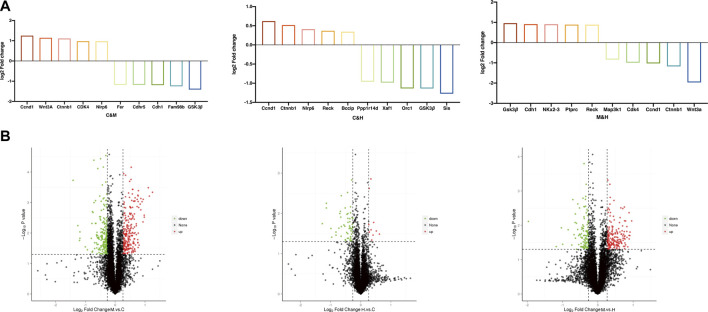
**(A)** TOP 5 DEPs. The project was selected to screen for upregulated expression proteins when FC ≥ 1.2, while *p* value ≤ 0.05, and for downregulated expression proteins when FC ≤ 0.83, while *p* value ≤ 0.05. **(B)** Volcano map of DEPs. The horizontal coordinate indicates the difference multiplicity of the differential protein (log2 value), and the vertical axis indicates the *p* value (−log10 value). Black represents proteins with no significant differences, red represents upregulated proteins, and green represents downregulated proteins. C: normal control; M: model control; H: HQT control, *n* = 3.

Protein difference analysis: In this study, a total of 6,889 proteins and 1,006 DEPs were identified from the colonic tissue of CAC mice, of which 546 proteins were upregulated, and 460 proteins were downregulated. Table 2 and Figure 6(B) reflect these changes. As shown in Figure 6(A), we screened the proteins after pairwise comparison and found that within the range of p < 0.05, the top five proteins significantly upregulated in the model group compared to the normal group were Ccnd1, WNT3A, Ctnnb1, CDK4, and Nlrp6; the top five proteins that were significantly downregulated were Fer, Cdhr5, Cdh1, Fam98b, and GSK3β. This indicates that the mice in the model group have elevated inflammatory factors, abnormal expression of WNT pathway‐related proteins, and excessive expression of cell cycle process-related factors. The top five proteins significantly upregulated in the HQT group compared to the model group were GSK3β, Cdh1, Map3k1, Ptprc, and Reck; the top five proteins that were significantly downregulated were NKx2‐3, Cdk4, Ccnd1, Ctnnb1, and WNT3A. It is shown that HQT can effectively increase the expression of immune antibodies and matrix metalloenzyme inhibitors in CAC mice and increase the expression of ECM related adhesion molecules and inhibit the inflammatory response‐related pathways with tumor cell differentiation and metastasis. The top five proteins significantly upregulated in the HQT group compared to the normal group were Ccnd1, Ctnnb1, Nlrp6, Reck, and Bccip, and the top five proteins significantly downregulated were Ppplr14d, Xaf1, Orc1, GSK3β, and Sis. The results revealed that the initiation of hypermethylation and DNA over‐replication occurred in mice under the conditions of simultaneous and continuous administration of AOM/DSS stimulation and HQT, but HQT played a somewhat inhibitory role.

**TABLE 2:** Protein number of total quantification. Compared samples: pairs of samples compared, as the former over the latter.

Category	Compared sample	*p* value	Protein
Upregulated	M&H	<0.01	21
		0.05 < and <0.01	190
	M&C	<0.01	100
		0.05 < and <0.01	223
	H&C	<0.01	2
		0.05 < and <0.01	10
Downregulated	M&H	<0.01	25
		0.05 < and <0.01	112
	M&C	<0.01	63
		0.05 < and <0.01	201
	H&C	<0.01	9
		0.05 < and <0.01	50
No significant	5883		
Total protein identified	6889		

C, normal control; M, model control; H, HQT control.

**FIGURE 7:**
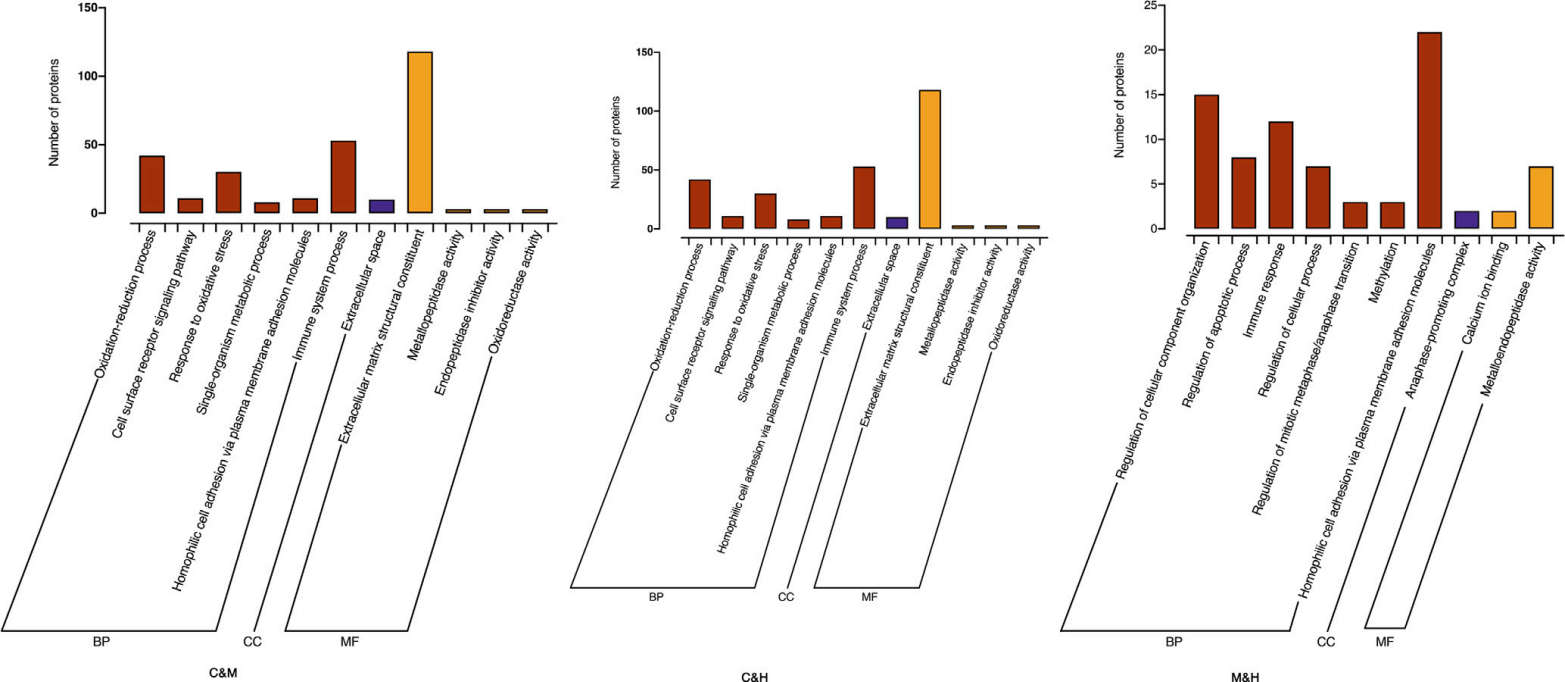
GO enrichment histogram, each presenting TOP20 (Adjusted*p*v<0.05, *n* = 3). C: normal control; M: model control; H: HQT control.

GO enrichment analysis: We used a corrected *p* value (Adjusted *p*v) < 0.05 to demonstrate the significant enrichment of GO functional entries in the differential proteins compared to all identified protein backgrounds in this study and made comparisons between groups. As shown in (Figure 7), GO function annotation showed that cellular component (CC) mainly included anaphase-promoting complex, extracellular space, and fibrinogen complex. Molecular functions (MF) were calcium ion binding, metalloendopeptidase activity, extracellular matrix structural constituent, endopeptidase inhibitor activity, oxidoreductase activity, receptor binding, and serine-type endopeptidase inhibitor activity. Biological process (BP) involved regulation of cellular component organization, regulation of cellular metabolic process, regulation of apoptotic process, immune response, immune system process, regulation of cellular process, regulation of mitotic metaphase/anaphase transition, methylation, homophilic cell adhesion *via* plasma membrane adhesion molecules, oxidation–reduction process, cell surface receptor signaling pathway, response to oxidative stress, single-organism metabolic process, protein polymerization, and response to stress. We suspect that repeated DSS stimulation causes the model mice to develop a response to oxidative stress and triggers their immune system, and that prolonged inflammatory infiltration produces inflammatory factors that stimulate cell surface receptor signaling pathways to affect homogenous cell adhesion *via* plasma membrane adhesion molecules. In contrast, HQT regulates the cellular composition and immune responses, regularly regulates the apoptotic process, and inhibits methylation and uncontrolled mitosis.

**FIGURE 8:**
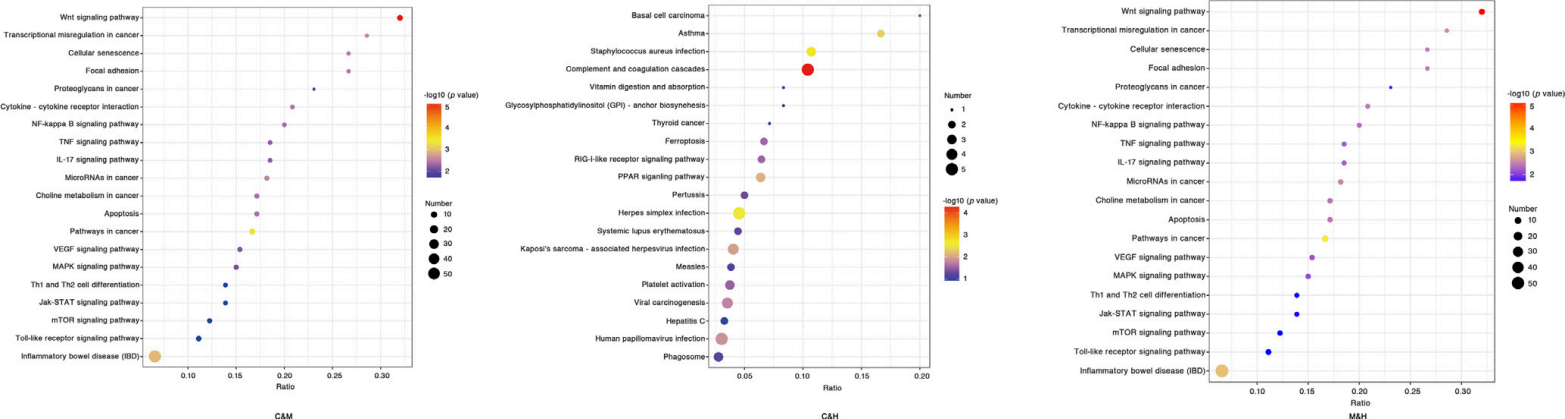
KEGG enrichment bubble chart. The horizontal coordinate is the ratio of the number of differential proteins in the corresponding pathway to the number of total proteins identified in the pathway, with larger values indicating higher levels of differential protein enrichment in the pathway. The color of the points in the graph represents the *p* value of the hypergeometric test, and the color ranges from blue to red; the redder color means the smaller value, indicating the greater reliability of the test and the more statistically significant. The size of the dots represents the number of differential proteins in the corresponding pathway, with larger dots indicating more differential proteins in that pathway. C: normal control; M: model control; H: HQT control, *n* = 3.

**FIGURE 9:**
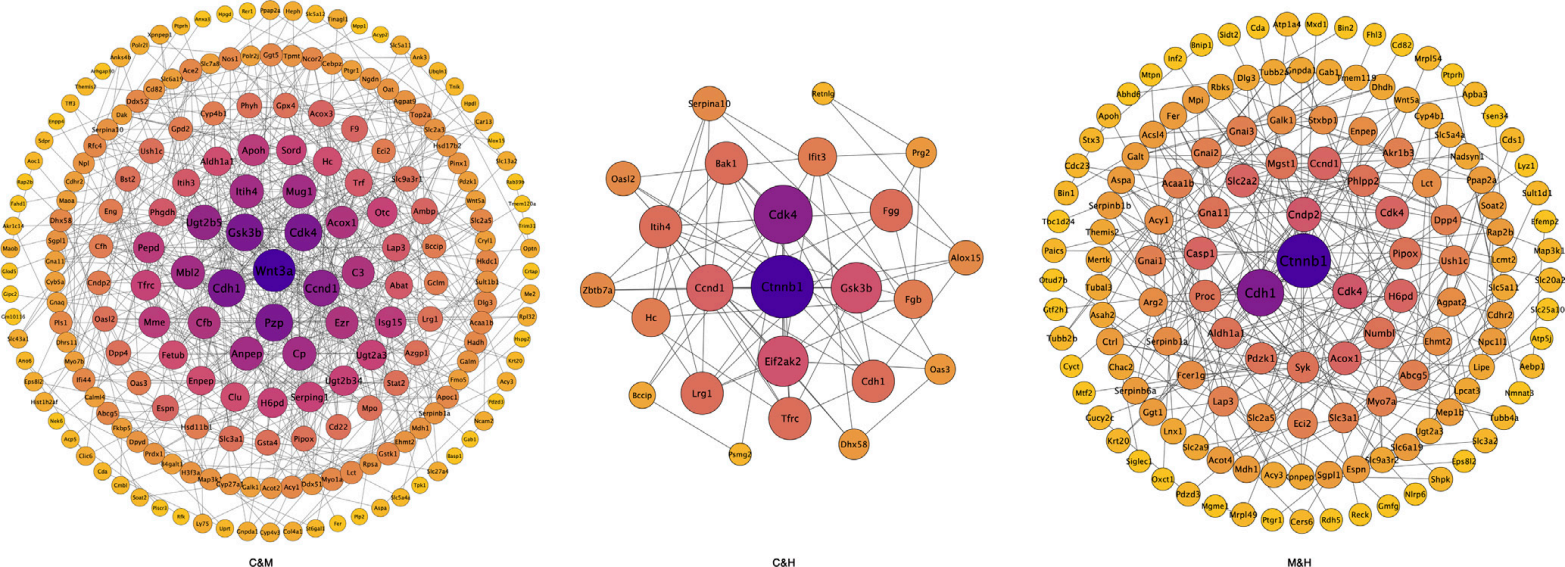
Protein–protein interactions network diagram. Each node in the interaction network represents a protein, the size of the node indicates the number of proteins interacting with it, with larger node indicating more proteins interacting with it, and the color of the node indicates the expression level of the protein, with darker colors indicating higher expression levels. C: normal control; M: model control; H: HQT control, *n* = 3.

KEGG enrichment analysis: We applied hypergeometric tests to find the pathways that were significantly enriched in the differential proteins compared with all identified proteins. The most prominent biochemical metabolic pathways and signal transduction pathways involved in differential proteins were identified by significant enrichment of pathways, and we produced the bubble plots as shown in Figure 8 with the uncorrected *p* value < 0.05 as standard, and only the TOP20 results were shown in the plots. The pathway enrichment results after screening with corrected (Adjusted*p*v) < 0.05 suggested that the previous studies have reported Wnt signaling pathway, NF-kappa B signaling pathway, p53 signaling pathway, and Jak-STAT signaling pathway that may be related to the occurrence of cancer were further validated in the present study. In addition, a number of other pathways were found to be significantly correlated that included pathways in cancer, cell cycle, focal adhesion, cellular senescence, Ppoteoglycans in cancer, cytokine–cytokine receptor interaction, chemical carcinogenesis, apoptosis, transcriptional misregulation in cancer, and microRNAs in cancer. Based on the aforementioned results, we have drawn the pathway diagram as shown in Figure 9.

Differential protein interactions analysis: We used the STRING-db protein interaction database for the interaction analysis of the identified proteins, extracted the sequences of the corresponding species/proximate species, and compared the sequences of the differential proteins with the extracted sequences by blast to obtain the corresponding interaction information as shown in Figure 9. We subsequently filtered the results of each two-by-two comparison by median and found that the main proteins with interactions were WNT3A, Gsk3*β*, Cdk4, Ccnd1, Cdh1, and Ctnnb1.

### Related Proteins and Signals Experimental Validation Analysis

**FIGURE 10:**
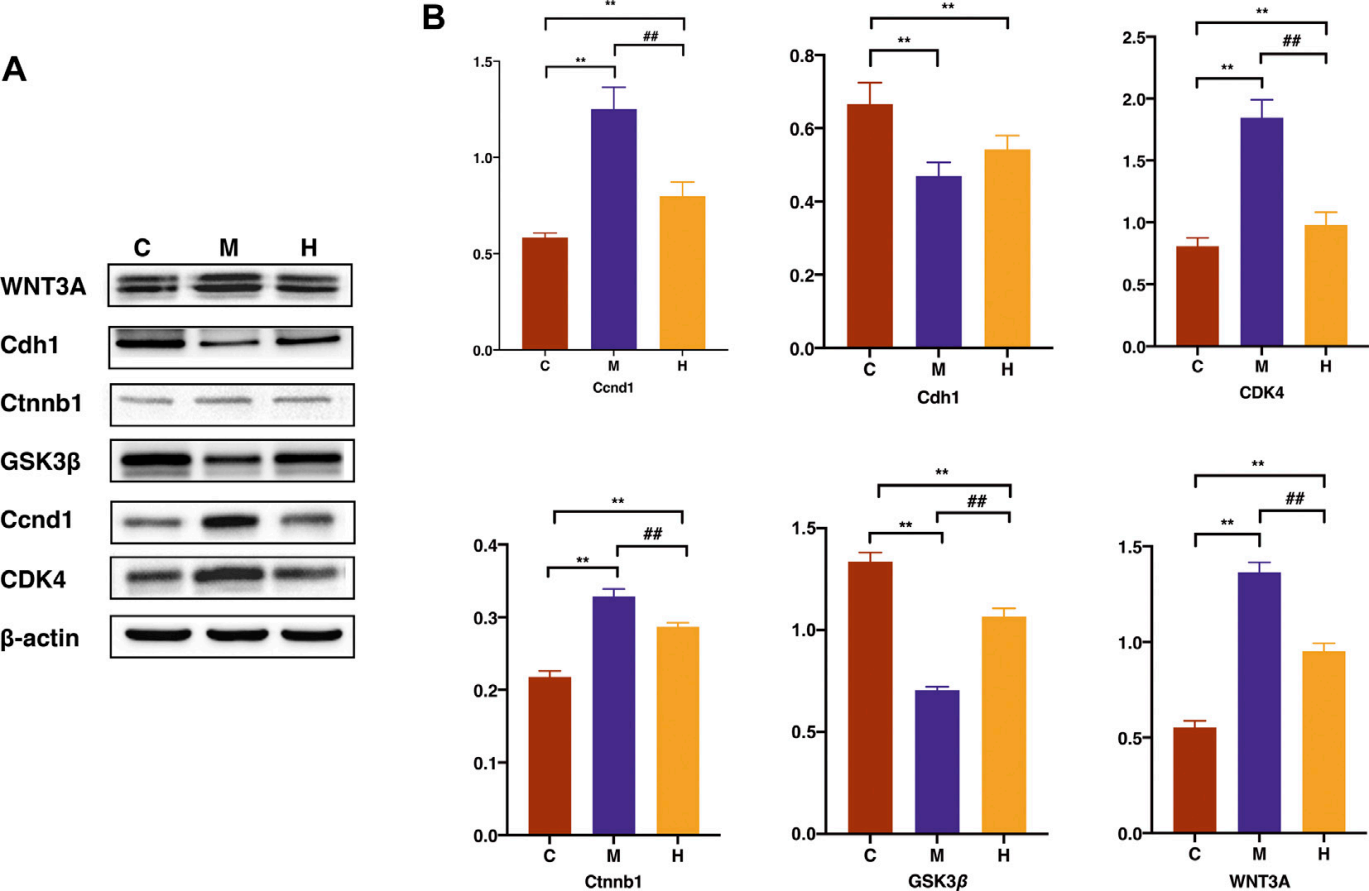
Effects of HQT on differential protein expression in CAC mice. **(A)** Western blot analysis of WNT3A、Cdh1、Ctnnb1、GSK3β、Ccnd1 and CDK4 in mice colon tissues. **(B)** Statistical analysis of WNT3A、Cdh1、Ctnnb1、GSK3β、Ccnd1 and CDK4 gray values. Data are represented as the mean±SD (*n* = 3, ¯x±s). *p<0.05, **p<0.01 vs. normal control; #*p*<0.05, ##*p*<0.01 vs. model control. C: Normal control; M: Model control; H: HQT control.

We have experimentally validated the differential protein expression profiles involved in the signaling pathways with the highest ranked relevance to tumorigenesis. The results shown in Figure 10A of the Western blot experiments on mice colon tissues were consistent with the trends of the analysis with our proteomic bioinformatics. As shown in the Figure 10B, the expression of proteins associated with tumourigenesis, namely, Ccnd1, Ctnnb1, WNT3A, and Cdk4 were significantly higher in the model group than in the normal group (*p* < 0.01) and rescued after the administration of HQT treatment. The expression of Gsk3β in the model group was significantly lower than that in the normal group (*p* < 0.01), and the expression increased after the administration of HQT (*p* < 0.01).

**FIGURE 11:**
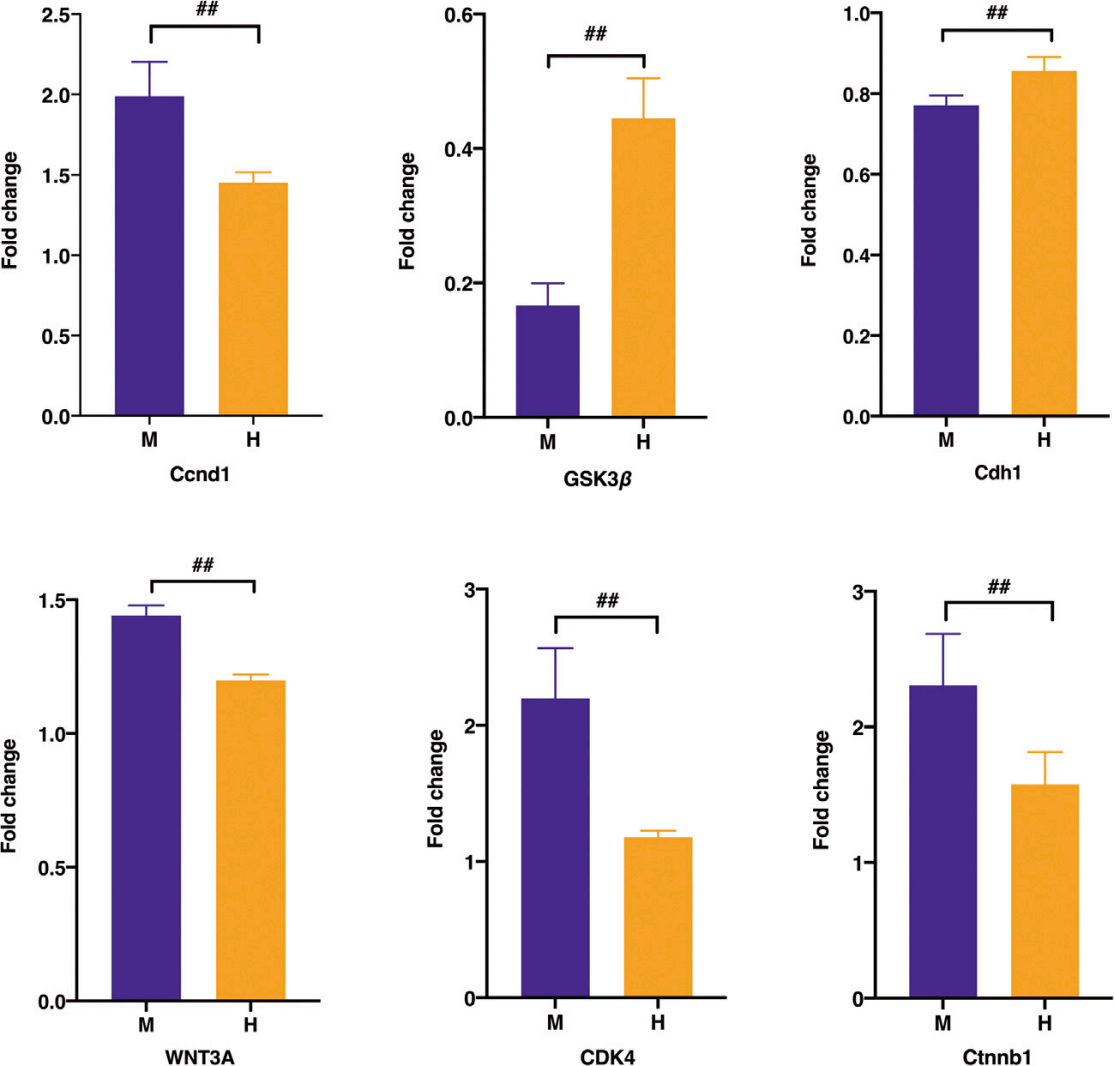
Effect of HQT on mRNA expression levels of the corresponding genes according to real-time PCR analysis. Data are represented as the mean ± SD (*n* = 5, ¯*x* ± *s*). ^*^ *p* < 0.05, ^**^ *p* < 0.01 vs. normal control; ^#^ *p* < 0.05, ^##^ *p* < 0.01 vs model control. C: normal control; M: model control; H: HQT control.

Regarding the expression of the differential protein at the RNA level, the real-time PCR test results in Figure 11 suggested that the expression of Ccnd1, Ctnnb1, WNT3A, and Cdk4 associated with uncontrolled proliferation was significantly higher than that in the normal group (*p* < 0.01), which was induced after the administration of HQT (*p* < 0.01). The expression of Gsk3β and Cdh1 was significantly weaker in the model group than that in the normal group (*p* < 0.01) and elevated after the administration of HQT.

## Discussion

From the molecular level, we combined quantitative proteomic assays based on CAC mice colon tissues and network target analysis to define HQT in the current study, which identified a list of differentially expressed proteins screened based on ploidy variation versus extent in the interaction network, including WNT3A, Ccnd1, Cdk4, Cdh1, Ctnnb1, and Gsk3*β*. These potential candidate protein targets were significantly associated with cell cycle and epithelial–mesenchymal transition according to the top ranked diseases and functions, all of which were related to tumorigenesis caused by unlimited cell proliferation. Moreover, we further analyzed the potential-regulating genes that were regarded as crucial components in some pathways to explore pharmacological mechanisms of HQT acting in the setting of CAC.

In order to enter the cell cycle and commence DNA replication, the cell must pass from the G1 phase into the S phase through strictly regulated restriction points (Murray, 1994). According to the “classical” cell cycle model, the G1/S transition starts at the early stage of G1 when the balance between mitogenic stimulation (*via* growth factor receptor activation) and inhibition tends to the former, triggering an increase in the levels of D-type cyclins (D1, D2, and D3). D-type cyclins bind to CDK4 or CDK6, and the cyclin-CDK complexes then enter the nucleus where they are phosphorylated by the CDK-activating kinase (CAK) complex. The enzymatic activities of CDK4 and CDK6 in the first gap phase (cycle G1) are controlled by D-type cyclins which respond to various extracellular signals, including stimulatory mitogens, inhibitory cytokines, differentiation inducers, cell–cell contacts, and other spatial cues (Malumbres and Barbacid, 2009). Dysregulation of the CDK/cyclin pathway permits unrestricted tumor growth by bypassing checkpoints that normally control cellular proliferation and genomic integrity (Garrett and Fattaey, 1999; Fry and Garrett, 2000; Hanahan and Weinberg, 2011). The literatures suggest that reducing the expression of Ccnd1 through antisense technology causes a concomitant decline in Ccnd1-dependent kinase activity and results in inhibition of tumor growth, abolition of tumorigenicity, or, in some instances, tumor cell death (Kozar, 2004; Asghar et al., 2015; Sherr et al., 2016; Goel et al., 2017). CAC model mice produce defective DNA repair and DNA damage checkpoints due to AOM injection, and genomic instabilities would normally make the deregulation of cell cycle control, causing cell division rapidly. Simultaneous administration of DSS stimulation to mice leads to persistent inflammatory infiltration, repeated tissue damage, and regeneration of tissue, in the presence of highly reactive nitrogen and oxygen species released from inflammatory cells, which interacts with DNA in proliferating epithelium resulting in permanent genomic alterations such as point mutations, deletions, or rearrangements. These unplanned proliferation and repair processes lead to CDKs dysregulation and transcriptional abnormalities. Experimental evidence suggests that HQT partially revert the transformed phenotype, perhaps by inhibiting cell cycle protein D-dependent kinase activity, leading to tumor growth inhibition. These results also confirm that Ccnd1/Cdk4 may indeed be a target for CAC treatment and allow a reasonable expectation that HQT, as an inhibitor of these enzymes, will produce a meaningful therapeutic response.

The cells in the body contact each other through subapical tight junctions, adherens junctions desmosomes at lateral surfaces, and scattered gap junctions at lateral surfaces, forming epithelial cell–cell junctions that are essential for epithelial integrity (Huang et al., 2012). Upon the initiation of EMT, these junctions are deconstructed, and the junction proteins are relocalized and/or degraded. During the destabilization of adherens junctions, epithelial cadherin (Cdh1) is cleaved at the plasma membrane and then degraded (Yilmaz and Christofori, 2009). Consequently, Ctnnb1 can no longer interact with Cdh1; it is either degraded or protected from degradation (for example, in response to the WNT signaling), so that it can play a role in transcription (Niehrs, 2012). As EMT progresses, the expression of junction proteins is transcriptionally repressed, which increases cell motility and stabilizes the loss of epithelial junctions (Peinado et al., 2007; De Craene and Berx, 2013). EMT is an integral part of development, and the processes underlying it are reactivated in wound healing, fibrosis, and cancer progression (Kalluri and Weinberg, 2009; Thiery et al., 2009; Chapman, 2011). Owing to the constitutively activated status and profound impact on EMT during cancer progression, the canonical WNT signaling pathway has become one of the most important molecular therapeutic targets for CAC (Feng et al., 2015). An elevated nuclear Ctnnb1 level is considered a hallmark of invasive CAC, mutations, or dysregulation of the Ctnnb1 destruction complex (APC, Axin2, CK1, and GSK-3*β*) that results in the activation of WNT-related targets, including c-myc, Ccnd1, MMP2, and MMP9, thereby promoting cell proliferative, invasive, and migratory potential (Khalil et al., 2009; Salmena et al., 2011; Guttman and Rinn, 2012; White et al., 2012). DSS is a heparin-like polysaccharide that is dissolved in the drinking water and inflicts CAC model mice colonic epithelial damage. During an episode of colitis, the mucosal adhesive gel-like coating barrier secreted by intestinal epithelial cup cells, which is used to maintain intestinal homeostasis and prevent the penetration of bacteria and pathogens, is disrupted. In response to tissue damage, a multifactorial network of chemical signals initiates and maintains a host response designed to “heal” the afflicted tissue. From the results of this work, we suspect that HQT maybe through stabilizing mutations in Ctnnb1 against degradation by the ubiquitin-proteasomal system, nuclear translocation is no longer be suppressed, and downstream target genes including c-Myc, Ccnd1, survivin, and porous metalloproteinase can be activated eventually. Additionally, GSK3*β* is no longer inhibited by WNT signaling due to the inhibition of Ctnnb1 accumulation in the nucleus and the inactivation of canonical WNT signaling, ultimately leading to a slowdown in the EMT process. Therefore, HQT may have the potential to target the WNT pathway and its related components as a therapeutic approach in CAC.

**FIGURE 12:**
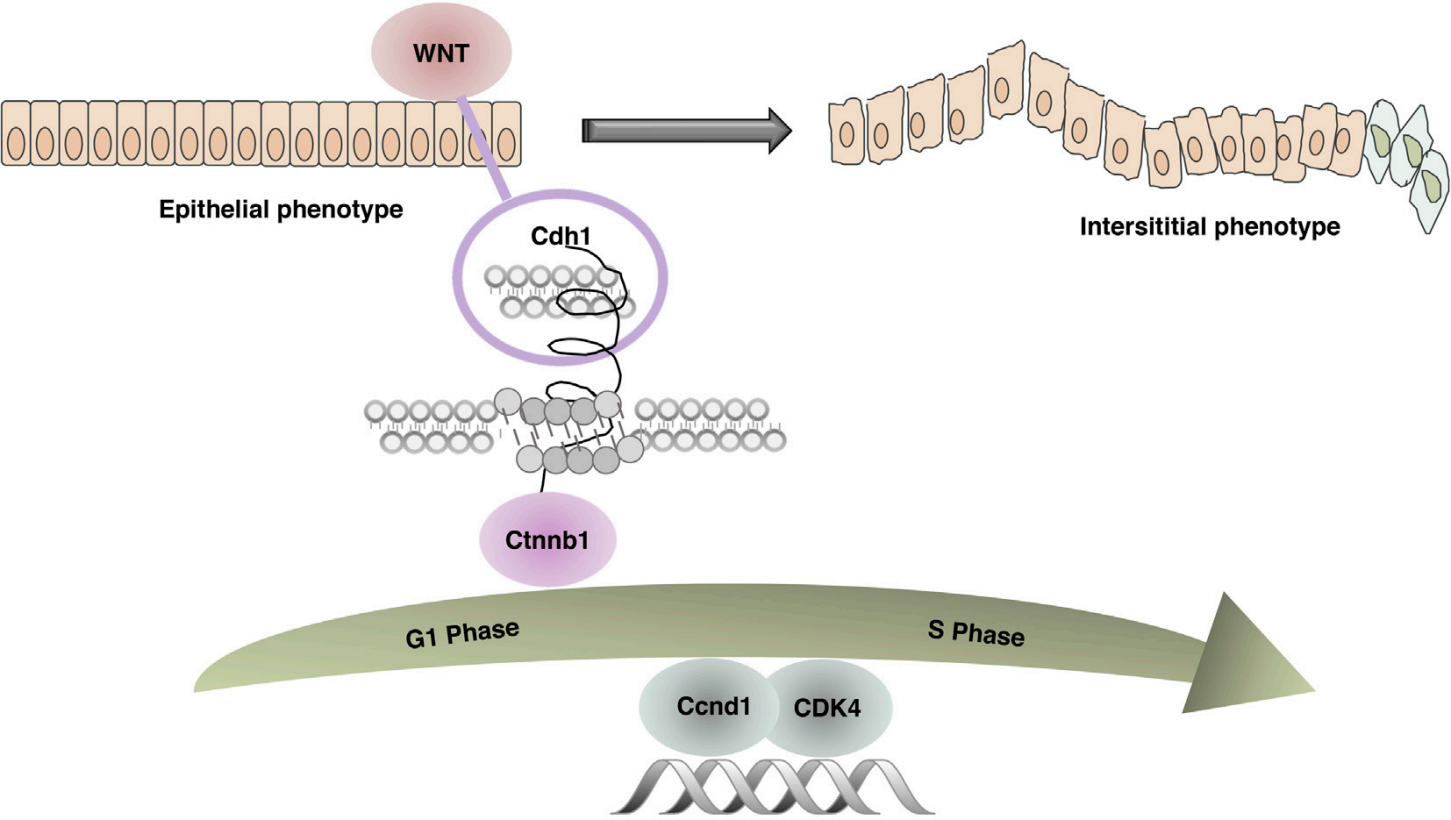
Related mechanism diagram. In the combined AOM/DSS-induced CAC mouse model, due to continuous inflammatory stimulation and repeated tissue damage, the WNT pathway was activated. Cdh1 and Ctnnb1 no longer interacted leading to unstable intercellular adhesion junctions, abnormal epithelial proliferation, accelerated tissue repair, dysregulation of cell cycle G1/S restriction point control, and abnormal expression of key regulators Ccnd1 and CDK4.

In conclusion, as we have illustrated in Figure 12, this comprehensive study provides compelling evidence that HQT can regulate tumorigenesis by modulating certain key targets screened by proteomics related to G1/S phase regulation and epithelial mesenchymal transition function. These new findings provide strong evidence that HQT may be an effective drug candidate for the treatment of CAC. This study has potential limitations; the effects estimates in the mice model are based on interventional and prospective observational studies. Admittedly, the main limitations of this study are the lack of species diversity in thesample and the small sample size due to time and effort constraints. In addition, we just describe the high-dose group to depict the exact efficacy of HQT but does not reflect an effective gradient of effective drug concentration. Considerably, more work will need to be carried out to update the dosing gradient in more depth, and we will subsequently employ a multiomics validation approach in conjunction with transcriptomics to further screen for potentially relevant action targets and adopt a molecular biology approach for experimental validation, with the aim of elucidating the specific mechanism of action of CAC pathogenesis and the pharmacological mechanism of action of HQT on CAC.

## Data Availability

The mass spectrometry proteomics data have been deposited to the ProteomeXchange Consortium (http://proteomecentral.proteomexchange.org) *via* the iProX partner repository with the dataset identifier PXD031696.
